# Supplementary material for: An optimal posttreatment surveillance strategy for cancer survivors based on an individualized risk-based approach
Source: Nat Commun. 2020 Aug 3;11:3872. doi: 10.1038/s41467-020-17672-w (PMC7400511; doi:10.1038/s41467-020-17672-w)
Supplement: Supplementary file 1 — Supplementary Information [file 41467_2020_17672_MOESM1_ESM.pdf]

Supplementary Information

An Optimal Post-treatment Surveillance Strategy for Cancer Survivors Based on an Individualized Risk-Based Approach

Guan-qun Zhou et al.

Supplementary Figures

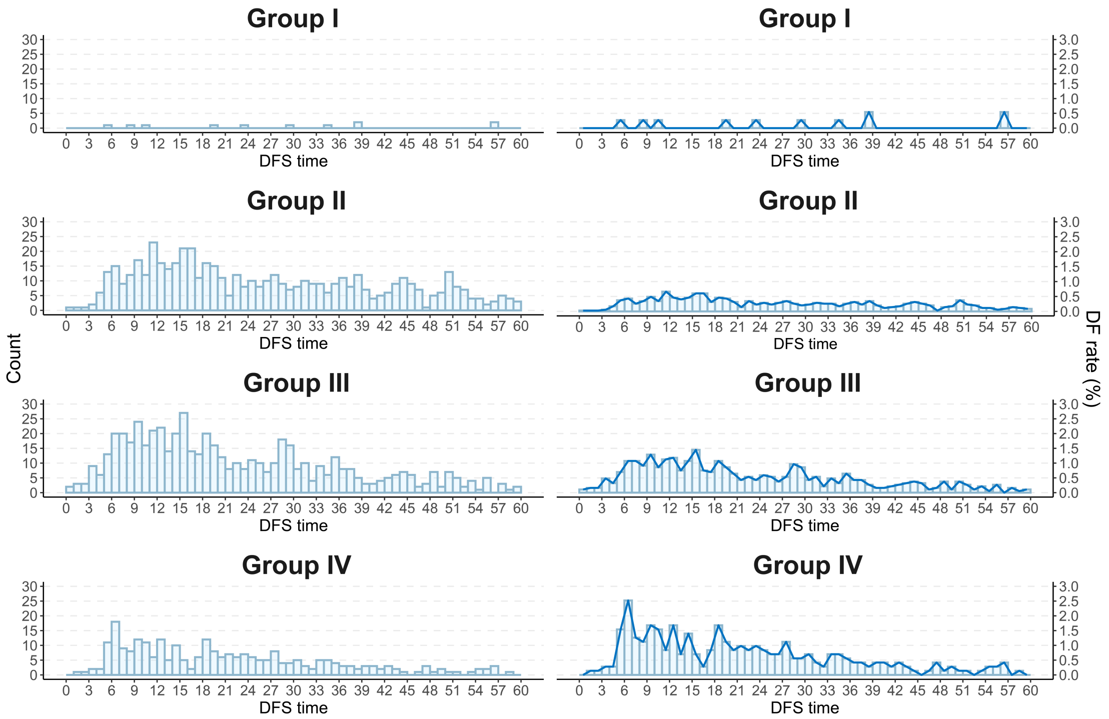

Supplementary Figure 1. The number of disease failure events and the crude incidence month by month in the different patient groups.

The number of disease failure events (left); the crude incidence (right). Number of patients: group I, n=367; group II, n=3,472; group III, n=1,863; group IV, n=714.

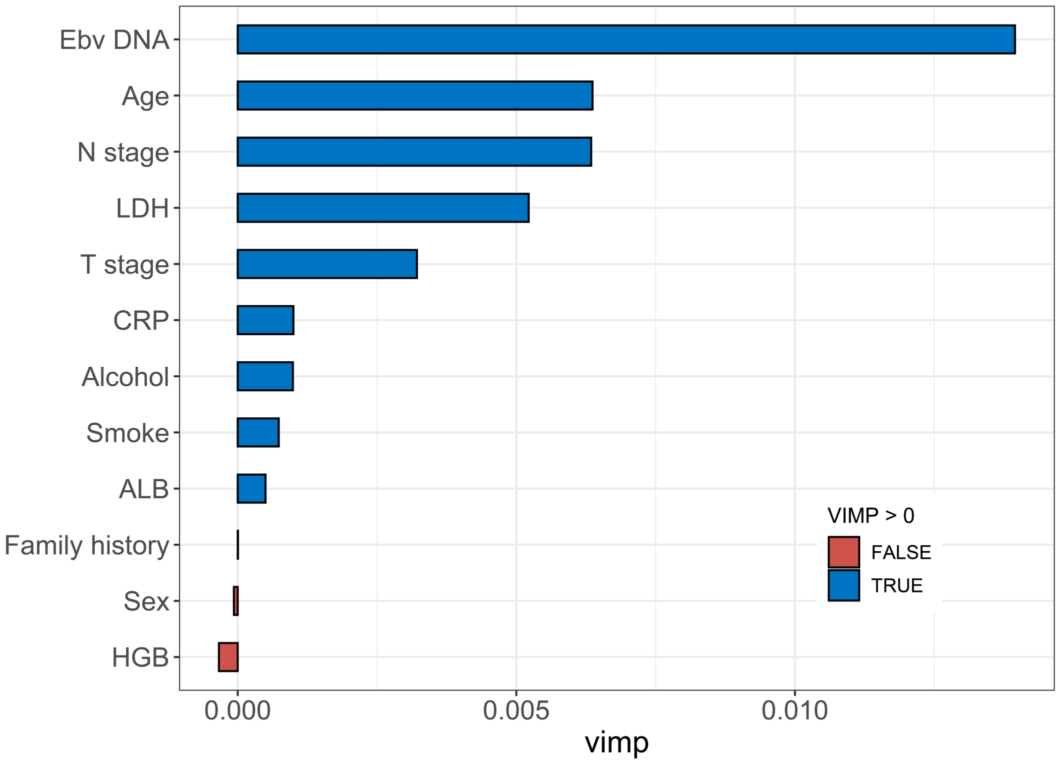

Supplementary Figure 2. The random forest variable importance for disease failure (n=6,416).

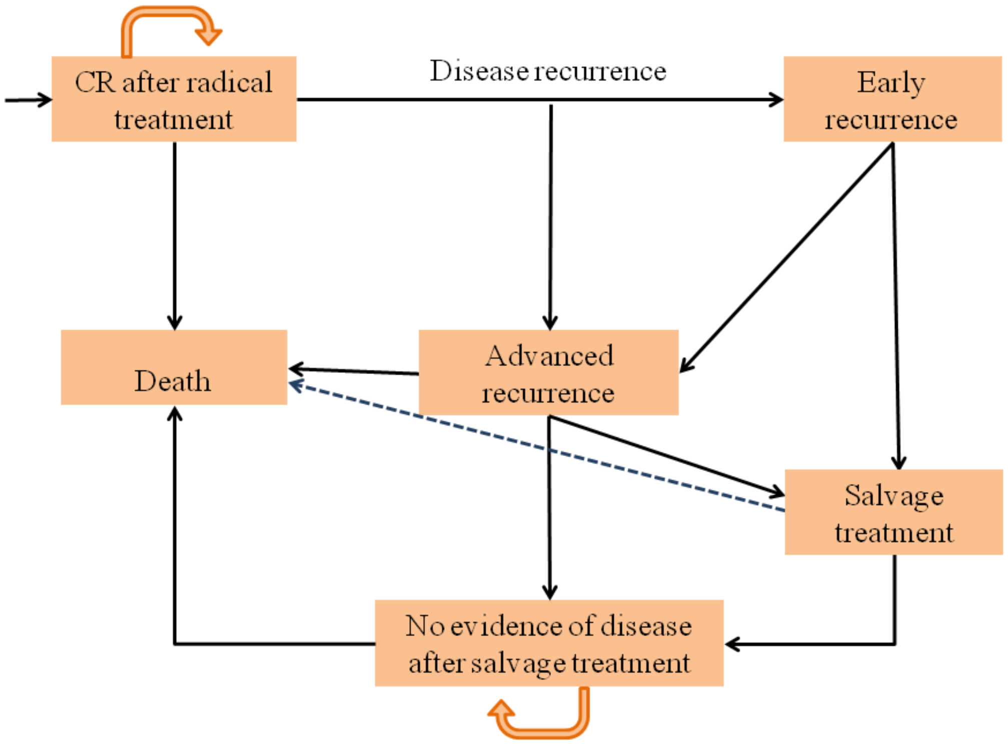

Supplementary Figure 3. Markov model of health states for patients with nasopharyngeal carcinoma after intensity-modulated radiotherapy.

CR, complete remission.

Supplementary Tables

Supplementary Table 1. Follow-up arrangements for a total of 13 visits in the patients of group III

Month	Probability per month	Follow-ups per month	Cumulative follow-ups	Follow-up scheduled
1	0	0	0	0
2	0.005526	0.071841	0.071841	0
3	0.005531	0.071897	0.143738	0
4	0.015512	0.20165	0.345388	0
5	0.011643	0.151363	0.49675	0
6	0.026589	0.345655	0.842405	1
7	0.045163	0.587116	1.429521	0
8	0.03742	0.486464	1.915985	1
9	0.033752	0.438777	2.354762	0
10	0.047574	0.618465	2.973227	1
11	0.031506	0.409576	3.382802	0
12	0.036826	0.478741	3.861543	1
13	0.041297	0.536865	4.398408	0
14	0.025025	0.325328	4.723736	1
15	0.03551	0.461636	5.185372	0
16	0.043128	0.560664	5.746036	1
17	0.021956	0.285426	6.031462	0
18	0.023226	0.301939	6.333401	0
19	0.036375	0.472877	6.806278	1
20	0.028038	0.364488	7.170766	0
21	0.020612	0.26796	7.438725	0
22	0.015779	0.205124	7.643849	0
23	0.017531	0.227904	7.871754	1

24	0.016015	0.208201	8.079955	0
25	0.019207	0.24969	8.329646	0
26	0.017201	0.223614	8.553259	0
27	0.013057	0.169739	8.722999	1
28	0.02016	0.262074	8.985073	0
29	0.02743	0.356595	9.341668	0
30	0.025725	0.334425	9.676093	1
31	0.01417	0.184205	9.860298	0
32	0.01573	0.204484	10.06478	0
33	0.006687	0.086932	10.15171	0
34	0.015404	0.200254	10.35197	0
35	0.011443	0.148765	10.50073	0
36	0.019128	0.248669	10.7494	1
37	0.012111	0.157448	10.90685	0
38	0.013166	0.171159	11.07801	0
39	0.008756	0.113822	11.19183	0
40	0.006459	0.083966	11.2758	0
41	0.00637	0.082813	11.35861	0
42	0.00701	0.09113	11.44974	0
43	0.009001	0.11701	11.56675	0
44	0.009886	0.128515	11.69526	1
45	0.011299	0.146887	11.84215	0
46	0.008599	0.111793	11.95394	0
47	0.003455	0.044921	11.99887	0
48	0.006271	0.081526	12.08039	0
49	0.012101	0.157313	12.2377	0
50	0.003482	0.04526	12.28296	0
51	0.15167	0.15167	12.43464	0
52	0.104245	0.104245	12.53888	1

53	0.046183	0.046183	12.58506	0
54	0.082151	0.082151	12.66721	0
55	0.038068	0.038068	12.70528	0
56	0.119827	0.119827	12.82511	0
57	0.028333	0.028333	12.85344	0
58	0.074934	0.074934	12.92838	0
59	0.031623	0.031623	12.96	0
60	0.04	0.04	13	0
Total	1.0000	13.0000	\	13

Supplementary Table 2. Cost-effectiveness analysis in the validation cohort

	Cost ($)	Incremental cost ($)	Effectiveness (QALYs)	Incremental effectiveness	ICER ($/QALY)
Patients in group I†					
The least intensive NCCN strategy	7,822	0	36.872	0	0
The moderately intensive NCCN strategy	8,629	807	37.356	0.484	1,667
The most intensive NCCN strategy	12,460	4,638	37.713	0.841	5,515
The RTOG strategy	8,672	850	37.357	0.485	1,753
The risk-based strategy※	7,793	-29	36.924	0.052	-558
Patients in group II†					
The least intensive NCCN strategy	10,881	0	32.398	0	0
The moderately intensive NCCN strategy	13,491	2,610	33.284	0.886	2,946
The most intensive NCCN strategy	18,520	7,639	34.076	1.678	4,552
The RTOG strategy	13,759	2,878	33.364	0.966	2,979
The risk-based strategy※	12,887	2,006	33.167	0.769	2,609
Patients in group III †					
The least intensive NCCN strategy	15,168	0	19.201	0	0
The moderately intensive NCCN strategy	17,412	2,244	19.798	0.597	3,759
The most intensive NCCN strategy	20,901	5,733	20.106	0.905	6,335
The RTOG strategy	17,573	2,405	19.904	0.703	3,421
The risk-based strategy※	17,174	2,006	19.847	0.646	3,105
Patients in group IV†					

The least intensive NCCN strategy	17,155	0	17.538	0	0
The moderately intensive NCCN strategy	19,231	2,076	18.052	0.514	4,039
The most intensive NCCN strategy	22,286	5,131	18.282	0.744	6,897
The RTOG strategy	18,908	1,753	18.061	0.523	3,352
The risk-based strategy※	18,903	1,748	18.098	0.56	3,121

† Patients were grouped according to TNM stages and EBV DNA.

※The dominant strategy.

Abbreviations: QALY, quality-adjusted life years; ICER, incremental cost-effectiveness ratio; NCCN, National Comprehensive Cancer Network; RTOG, Radiation Therapy Oncology Group.

Supplementary Table 3. Baseline clinical estimates for Markov model construction

	Estimate (%)	Reference
Initial distribution of patients with recurrence		Assumption
Proportion of early stage in distant metastasis	80	
Proportion of early stage in local recurrence	90	
Proportion of early stage in regional recurrence	90	
Probability of transformation to advanced stage if early recurrence if not detected within a month		Assumption
for distant metastasis	20	
for local recurrence	10	
for regional recurrence	10	
Survival probabilities given disease recurrence after treatment		
5-year survival given early distant metastasis	42	27, 29
5-year survival given advanced distant metastasis	14	27, 29
5-year survival given early local recurrence	48	6, 30
5-year survival given advanced local recurrence	18	6, 30
5-year survival given early regional recurrence	86	26, 28
5-year survival given advanced regional recurrence	50	26, 28
Survival probability given undetected disease recurrence		Assumption
2-year survival given early distant metastasis	0	
2-year survival given advanced distant metastasis	0	

2-year survival given early local recurrence	30	
2-year survival given advanced local recurrence	10	
2-year survival given early regional recurrence	30	
2-year survival given advanced regional recurrence	10	
Survival probability given disease recurrence that is not detected within 3 years	0	Assumption
Model costs ($)		From the Medical Insurance Administration Bureau
Cost of routine follow-up	255	
Cost of treatment for distant metastasis	28,986	
Cost of treatment for local recurrence	13,333	
Cost of treatment for regional recurrence	11,014	
Model utilities		24, 25, 31
No evidence of recurrence	0.8	
Distant metastasis	0.4	
Local recurrence	0.35	
Regional recurrence	0.4	
CR after treatment for distant metastasis	0.55	
CR after treatment for local recurrence	0.5	
CR after treatment for regional recurrence	0.6	

Abbreviations: CR, complete remission.

Supplementary Methods

Plasma EBV DNA quantification

Peripheral blood (3 ml) were obtained in an EDTA tube and centrifuged at 1600 x g for 15 min to isolate plasma and peripheral blood cells. Viral DNA was extracted by the QIAamp Blood Kit (Qiagen, Hilden, Germany). A total of 500 μl plasma sample was used for DNA extraction per column and a final elution volume of 50 μl was utilized to elute the DNA. Plasma EBV DNA concentrations were measured by real-time quantitative polymerase chain reaction (PCR) assay of the BamHI-W region of the EBV genome[1]. The sequences of the forward and reverse primers were: 5`-GCCAG AGGTA AGTGG ACTTT-3` and 5`-TACCA CCTCC TCTTC TTGCT-3` respectively. And the probe was a dual fluorescently-labelled oligomer 5`-(FAM) CACAC CCAGG CACAC ACTAC ACAT (TAMRA)-3`. An Applied Biosystems 7700 Sequence Detector was used for amplifications, which was further analyzed using the Sequence Detection System software (version 1.6.3) of Applied Biosystems (Foster City, CA). The plasma EBV DNA quantifications were calculated using the following equation: $C=Q\times(VDNA/VPCR)\times(1/VEXT)$; C is the target concentration in plasma (copies/ml), Q is the target quantity (copy number) determined by PCR, VDNA is the total volume of DNA obtained after extraction (typically 50 μl/Qiagen extraction), VPCR is the volume of DNA solution used for PCR (typically 2 μl) and VEXT is the volume of plasma extracted (typically 0.5 ml).

References

1. Cohen EE*, et al.* American Cancer Society Head and Neck Cancer Survivorship Care Guideline. *CA: a cancer journal for clinicians* **66**, 203-239 (2016).
