# Supplementary material for: An optimal posttreatment surveillance strategy for cancer survivors based on an individualized risk-based approach
Source: Nat Commun. 2020 Aug 3;11:3872. doi: 10.1038/s41467-020-17672-w (PMC7400511; doi:10.1038/s41467-020-17672-w)
Supplement: Supplementary file 3 — Description of Additional Supplementary Files [file 41467_2020_17672_MOESM3_ESM.pdf]

Description of Additional Supplementary Files

File Name: Supplementary Software 1
Description: Contains a README.docx file, R codes and simulated datasets as demos.
