# Supplementary material for: An optimal posttreatment surveillance strategy for cancer survivors based on an individualized risk-based approach
Source: Nat Commun. 2020 Aug 3;11:3872. doi: 10.1038/s41467-020-17672-w (PMC7400511; doi:10.1038/s41467-020-17672-w)
Supplement: Supplementary file 4 — Supplementary Software 1 [file 41467_2020_17672_MOESM4_ESM.zip › Supplementary Software/README.docx]

**Establish the Optimal Post-treatment Surveillance Strategy for Cancer Survivors Based on the Individualized Risk-Based Approach**

**System Requirements**

**Hardware Requirements**

The code requires a standard computer with enough RAM to support the software operations.

**Software Requirements**

*OS Requirements*

The code has been tested on the following systems:

Windows10

MacOS: Mojave (10.14.6)

*Software*

R Version 3.6.1 (https://www.r-project.org)

*R Packages Used*

readr

readxl

plyr

randomForestSRC

ggplot2

**Installation Guide**

*R Software*

The R software can be downloaded from <https://www.r-project.org>, with the installation guide available at <https://cran.r-project.org/doc/FAQ/R-FAQ.html#How-can-R-be-installed_003f>. The download and installation time depend on individuals’ internet and computers condition, which would typically finish in about 1 minute.

*R Packages*

To install the required R packages, just enter the following code in the R Console: install.packages(c("readr", "readxl", "plyr", "randomForestSRC", "ggplot2")), and the packages would be installed in about 40 seconds.

**Instructions and Demo**

The source code we provided utilized the demo data as examples to show the detailed analysis of the process and the results. The source code and the demo were given in the supplementary files, and the expected run time for the demo is about 2 minutes.
