# Supplementary material for: An optimal posttreatment surveillance strategy for cancer survivors based on an individualized risk-based approach
Source: Nat Commun. 2020 Aug 3;11:3872. doi: 10.1038/s41467-020-17672-w (PMC7400511; doi:10.1038/s41467-020-17672-w)
Supplement: Supplementary file 5 — Reporting Summary [file 41467_2020_17672_MOESM5_ESM.pdf]

nature research

Corresponding author(s): Ying Sun & Jin-hui Liang

Last updated by author(s): Jun 22, 2020

Reporting Summary

Nature Research wishes to improve the reproducibility of the work that we publish. This form provides structure for consistency and transparency in reporting. For further information on Nature Research policies, see our Editorial Policies and the Editorial Policy Checklist.

Statistics

For all statistical analyses, confirm that the following items are present in the figure legend, table legend, main text, or Methods section.

n/a	Confirmed	
☐	☒	The exact sample size (*n*) for each experimental group/condition, given as a discrete number and unit of measurement
☐	☒	A statement on whether measurements were taken from distinct samples or whether the same sample was measured repeatedly
☒	☐	The statistical test(s) used AND whether they are one- or two-sided<br>*Only common tests should be described solely by name; describe more complex techniques in the Methods section.*
☐	☒	A description of all covariates tested
☐	☒	A description of any assumptions or corrections, such as tests of normality and adjustment for multiple comparisons
☒	☐	A full description of the statistical parameters including central tendency (e.g. means) or other basic estimates (e.g. regression coefficient) AND variation (e.g. standard deviation) or associated estimates of uncertainty (e.g. confidence intervals)
☒	☐	For null hypothesis testing, the test statistic (e.g. *F*, *t*, *r*) with confidence intervals, effect sizes, degrees of freedom and *P* value noted<br>*Give P values as exact values whenever suitable.*
☒	☐	For Bayesian analysis, information on the choice of priors and Markov chain Monte Carlo settings
☐	☒	For hierarchical and complex designs, identification of the appropriate level for tests and full reporting of outcomes
☒	☐	Estimates of effect sizes (e.g. Cohen's *d*, Pearson's *r*), indicating how they were calculated

Our web collection on statistics for biologists contains articles on many of the points above.

Software and code

Policy information about availability of computer code

Data collection	Excel.
Data analysis	R version 3.4.3 random Forest SRC package (Version 2.6.1, Ishwaran et al., 2018). Detailed code and associated instructions are provided in the Supplementary Software file.

For manuscripts utilizing custom algorithms or software that are central to the research but not yet described in published literature, software must be made available to editors and reviewers. We strongly encourage code deposition in a community repository (e.g. GitHub). See the Nature Research guidelines for submitting code & software for further information.

Data

Policy information about availability of data

All manuscripts must include a data availability statement. This statement should provide the following information, where applicable:

- Accession codes, unique identifiers, or web links for publicly available datasets
- A list of figures that have associated raw data
- A description of any restrictions on data availability

The data of patient characteristics, therapies and survival information have been deposited in the Research Data Deposit public platform (www.researchdata.org.cn, accession code: RDDA2018000934). All the other data of this study are available within the Article, Supplementary Information or from the corresponding author upon reasonable request.

Field-specific reporting

Please select the one below that is the best fit for your research. If you are not sure, read the appropriate sections before making your selection.

- [x] Life sciences
- [ ] Behavioural & social sciences
- [ ] Ecological, evolutionary & environmental sciences

For a reference copy of the document with all sections, see nature.com/documents/nr-reporting-summary-flat.pdf

Life sciences study design

All studies must disclose on these points even when the disclosure is negative.

Sample size	In the training cohort, we adopted an NPC-specific database from the well-established big-data intelligence platform, and identified 7,108 patients with histologically proven, non-disseminated NPC, diagnosed between Apr 2009 and Oct 2014. In this study, we identified eligible patients from the extracted cohort. In the external validation cohort, we included we included 627 patients from Wuzhou Red Cross Hospital. Thus, a total of 7,043 patients were included in this study. To ensure that the sample size is sufficient in the study, we hypothesized the differences in mean delay in detection ranging from 0.1 to 1 month (step size, 0.1) with standard deviation ranging from 1 to 5 (step size, 1). With a 2-sided α=0.05, the sample size of our study resulted in power ranging from 89.3% to 100%, which is all larger than 80% and thus indicates that the sample size in the study is sufficient.
Data exclusions	The data exclusion criteria were pre-established before the study analyses. A total of 691 patients were excluded for the following reasons: insufficient information including smoke, alcohol, family history, EBV DNA, hemoglobin (HGB), albumin (ALB), C-Reactive protein (CRP), lactate dehydrogenase (LDH) (n= 593), fewer than 90 days of follow-up (n=66), development of metastatic disease within 30 days of radiotherapy (n=33).
Replication	Analysis progress is detailed in the Methods section in the article. And the code utilized during the analysis and associated instructions with a demo are provided in the Supplementary Software file to guarantee the reproducibility of the experimental findings. All attempts at replication were successful.
Randomization	The allocation is not randomized. To control for potential confounding covariates, we used the random survival forest.
Blinding	Blinding was not relevant for our study as there was no pre-defined groupings.

Reporting for specific materials, systems and methods

We require information from authors about some types of materials, experimental systems and methods used in many studies. Here, indicate whether each material, system or method listed is relevant to your study. If you are not sure if a list item applies to your research, read the appropriate section before selecting a response.

Materials & experimental systems

n/a	Involved in the study
x	Antibodies
x	Eukaryotic cell lines
x	Palaeontology and archaeology
x	Animals and other organisms
	x Human research participants
x	Clinical data
x	Dual use research of concern

Methods

n/a	Involved in the study
x	ChIP-seq
x	Flow cytometry
x	MRI-based neuroimaging

Human research participants

Policy information about studies involving human research participants

Population characteristics	The median age for the group was 45 years (interquartile range [IQR], 38 to 53 years). There were 4,753 male and 1.663 female. A total of 6,380 patients (99.4%) were diagnosed as histological WHO Type IIa/IIb. T1, T2, T3 and T4 NPC was present in 10.4%, 17.8%, 47.5% and 24.3% of the study patients, respectively. The distribution of N category was N0 (12.8%), N1 (46.2%), N2 (30.8%), N3 (10.3%). For the whole group, 5,678 patients (88.5%) were treated with chemo-radiotherapy and 738 patients were treated with radiotherapy alone.
Recruitment	We adopted an NPC-specific database from the well-established big-data intelligence platform, and identified 7,108 consecutive patients with histologically proven, non-disseminated NPC, diagnosed between Apr 2009 and Oct 2014. A total of 691 patients were excluded for the following reasons: insufficient information including smoke, alcohol, family history, EBV DNA, hemoglobin (HGB), albumin (ALB), C-Reactive protein (CRP), lactate dehydrogenase (LDH) (n= 593), fewer than 90 days of follow-up (n=66), development of metastatic disease within 30 days of radiotherapy (n=33). In the external validation cohort, we included we included 627 patients with histologically proven, non-disseminated NPC from Wuzhou Red Cross Hospital. Thus, a total of 7,043 patients were included in this study. The retrospective nature of this study may introduce potential biases because of the missing data and the populational heterogeneity. However, the large cohort derived from the NPC-specific database, which is a well-established big data intelligence platform-based clinical research system, and the covariate-adjusted survival probabilities using the random survival forest model might reduce biases. In addition, the external validation further advocate the robustness and the general applicability of our findings.
Ethics oversight	The institutional ethics committees of Sun Yat-Sen University Cancer Centre and Wuzhou Red Cross Hospital approved the study protocol and waived the requirement for informed consent given the retrospective nature of the study.

Note that full information on the approval of the study protocol must also be provided in the manuscript.
